# Match Loads May Predict Neuromuscular Fatigue and Intermittent-Running Endurance Capacity Decrement after a Soccer Match

**DOI:** 10.3390/ijerph192215390

**Published:** 2022-11-21

**Authors:** Diego Marqués-Jiménez, Julio Calleja-González, Iñaki Arratibel-Imaz, Nicolás Terrados

**Affiliations:** 1 Valoración del Rendimiento Deportivo, Actividad Física y Salud y Lesiones Deportivas (REDAFLED), Department of Didactics of Musical, Plastic and Corporal Expression, Faculty of Education, University of Valladolid, 42004 Soria, Spain; 2 Department of Physical Education and Sports, Faculty of Education & Sport, University of the Basque Country, 01007 Vitoria-Gasteiz, Spain; 3 Sports Medicine Center Tolosa Kirol Medikuntza, 20400 Tolosa, Spain; 4 Regional Unit of Sports Medicine of the Principality of Asturias (URMD), 33401 Avilés, Spain; 5 Health Research Institute of the Principality of Asturias (ISPA), 33011 Oviedo, Spain

**Keywords:** fatigue, intermittent running, match loads, neuromuscular, soccer

## Abstract

How the match-derived load metrics relate to post-match fatigue in soccer is scarcely researched. Thus, the aim of this study was to determine the associations between soccer match-related internal and external loads, neuromuscular performance decrease and intermittent-running endurance capacity decrement immediately post-match. Vertical jump (countermovement jump), straight-line sprinting (10- and 20-m sprint), change of direction ability (T-test) and intermittent-running endurance capacity (YO-YO intermittent recovery level 2) were measured one day before and immediately after a friendly match in male soccer players. During the match, players’ internal and external loads were also monitored, including heart rate-derived indices, total distance at various speed thresholds, average running velocity, maximal running velocity, number of sprints and number of accelerations and decelerations at various intensity thresholds. The results show that match-induced fatigue was reflected on neuromuscular performance and intermittent-running endurance capacity immediately post-match (*p* < 0.05). The quantification of percentage change of match external-load metrics, particularly accelerations and decelerations, provides a useful non-invasive predictor of subsequent neuromuscular fatigue status in soccer players immediately post-match (*p* < 0.05). However, only internal load metrics present a practical application for predicting intermittent-running endurance capacity impairment (*p* < 0.05). In summary, internal and external load metrics may allow for predicting the extent of acute fatigue, and variability between halves may represent a valuable alternative to facilitate the analysis of match-related fatigue both for research and applied purposes.

## 1. Introduction

Soccer match load alters the biochemical milieu, increases muscle damage and causes glycogen depletion due to its high-intensity intermittent nature [1,2], leading to neuromuscular fatigue (NMF) and physical performance impairment up to 72–96-h post-match [1]. While previous studies concerning the etiology of fatigue after matches have mainly focused on peripheral impairments, recent studies suggest that competitive match play also elicits perturbations in the functioning of the central nervous system which take days to recover [3].

Although challenging due to the multi-factorial nature of fatigue, an effective neuromuscular performance and intermittent-running endurance capacity monitoring could provide insights into soccer players’ physiological status, which may then further help in the attempt to control the athletes’ adaptations and reduce the susceptibility of injury and illness [4]. On one hand, the gold standard assessment of NMF requires magnetic or electrical nerve stimulation, but this approach is impractical in the applied sport setting [5]. To overcome this limitation, NMF can be quantified via countermovement jump performance (CMJ). It has been suggested that its high repeatability and sensitivity prove its usefulness as a fatigue marker [6], even compared with the sprint performance [7]. On the other hand, intermittent-running endurance capacity can be measured using YO-YO intermittent recovery (YO-YO IR) tests, a simple method for examining a player’s capacity to perform repetitive high-intensity aerobic exercise [8]. The YO-YO IR1 test has been suggested as a method primarily to test endurance capacity, whereas the YO-YO IR2 test was introduced to determine the ability to repeatedly perform intense exercise with a high anaerobic energy contribution [8]. 

Monitoring fatigue status through neuromuscular performance and intermittent-running endurance capacity is, however, time-consuming and may involve performance tests or invasive measurements that are difficult to implement in the daily training routine. Consequently, fatigue monitoring requires practical approaches using data derived in training sessions and the development of tools to enable the simultaneous, instantaneous and non-invasive capture of multiple sources of information during match play [9]. In this scenario, time–motion analysis using wearable tracking devices is now commonplace in sport research and practice [10], and requires minimal player involvement and additional assessments [11]. The use of such technology in team sports has increased in recent years, both in research and in practical applications [10]. These devices provide large amounts of external load data, such as total distance covered (TD), distance covered at different speed intensities, count and distance on accelerations and decelerations at different intensity thresholds or number of physical contacts, among others. These derived physical performance metrics can be used to lead the decision-making of athlete training direction and to determine when to apply the next training stimulus [10]. Moreover, they could also be used to assess fatigue level, to help in planning the recovery process and in injury prevention [10].

Accordingly, some associations have been made between match activity metrics and fatigue measures. A recent systematic review analyzed the relationships between match external-load metrics and acute (up to 24-h) and residual (up to 72-h) fatigue markers. The findings indicate that very high-intensity running activities (>5.5 ms^−1^) are strongly correlated with NMF based on the CMJ performance, but only during the first 24-h after a match [12]. Yet, how the match-derived internal load variables relate to post-match fatigue is scarcely researched. Studies investigating several external load variables and their effect on sprint or intermittent running performance are also needed [11]. Therefore, the main aim of the present study was to determine the associations between soccer match-related internal and external loads, neuromuscular performance decrease and intermittent-running endurance capacity decrement immediately post-match. This investigation approach could maximize the opportunity to predict short-term neuromuscular and intermittent running performance impairments from internal and external loads, identifying which metrics are the most relevant in fatigue player management.

## 2. Materials and Methods

### 2.1. Participants

Ten male semiprofessional soccer players (age = 22.92 ± 2.38 years; height = 176.5 ± 4.84 cm; body mass = 71.18 ± 5.7 kg) from a team participated in this study. The team usually trained four days per week. Training sessions included a higher volume of small-sided games (SSGs) than unopposed technical or tactical drills and fitness training. All participants were healthy and were informed of the procedures, methods, benefits and possible risks related to the study. The procedures were in accordance with the ethical standards on human experimentation established in the Declaration of Helsinki (1964), updated in Fortaleza (2013). All players signed a statement of informed consent to participate in the study, approved by the Ethics Committee of the University of the Basque Country.

### 2.2. Experimental Design

All the procedures were carried out in-season (week without official competition). Players were requested to avoid physical exercise in the 48-h preceding baseline measurements. Instructions for each test were provided in a familiarization session four weeks before the baseline measurements.

The friendly match was played in the morning (11:00 am, 15.2 °C, 77.2% relative humidity) on a turf surface. The match lasted 90 min, and each player played the full time (no substitutions were permitted). The 4-4-2 formation was used by the team involved in the current study, so internal and external loads were obtained from all playing positions of this formation. Each player played the full match in the same position. Therefore, plausible positional differences did not influence the predictive capacity of external or internal load measurements. The players were tested in a covered pavilion to maintain similar environmental conditions during testing procedures. They did not accomplish the test protocol wearing soccer boots.

One day before the match, data of neuromuscular performance and intermittent-running endurance capacity were collected. They were considered as baseline measurements (pre-match). The test battery started with short anaerobic tests and ended with strenuous tests, and was carried out in the following order: vertical jump (CMJ), straight-line sprinting (10- and 20-m sprint), change of direction ability (COD; T-test) and intermittent-running endurance capacity (YO-YO IR2). Participants performed three CMJ, two sprints over 20-m (10-m data was also obtained), two T-tests and the YO-YO IR2. In the straight-line sprinting and T-test, the players decide to start when ready, without any reaction time bias. All players repeated the attempts with a 3-min recovery period between them, while a 5-min recovery period was applied between tests. The time needed to test all of the players (players walking to the testing station, getting in order, etc.) helped to provide the rest period of several minutes for each player. All measurements were performed again immediately after the match (the time between the end of the match and the CMJ was ≃10-min). The same testing order was also applied.

A standard warm-up was conducted before physical performance tests. It included jogging, dynamic stretching, jumps, COD drills, acceleration runs and sprints. Participants also performed the standard warm-up before the match. 

### 2.3. Physical Performance Measurements

The CMJ performance was evaluated using the variable jump height, which has shown excellent test–retest reliability for measuring NMF, with an intraclass correlation coefficient (ICC) of 0.88 and a coefficient of variation (CV) of 4.8% in elite youth soccer players [13]. Jump height was measured using a platform with infrared rays (Optojump Next, Microgate^®^, Bolzano, Italy). The mean values of the three jumps were used for further statistical analysis, because the average value is more sensitive than the highest one in identifying fatigue [14]. 

Three pairs of photoelectric cells (Microgate Racetime2, Microgate^®^, Bolzano, Italy) placed 0.4 m above the ground were used to evaluate the straight-line sprinting performance. The players started when they were ready from a standing position of 0.5 m away from the first photocell gate and sprinted to the finish line (20-m) where the last photocell had been located (finish time). The mean time of both attempts were used for further statistical analyses.

The COD performance was evaluated by the T-test, which was administered using the protocol outlined by Semenick [15]. It has shown high reliability (*r* = 0.89) in male soccer players [16]. The COD performance was measured using photocell gates (Microgate Racetime2, Microgate^®^, Bolzano, Italy). One pair of photoelectric cells was set 0.4 m above the ground and located 3 m apart, with each cell facing each other on either side of the starting line. The clock started when players passed the photoelectric cells, and the clock stopped the instant they again crossed the photoelectric cells. The mean time of both attempts were used for further statistical analyses.

Intermittent-running endurance capacity was measured using the YO-YO IR2 as described by Krustrup et al. [17]. It has a high reproducibility and sensitivity, so individual differences and changes in the performance of players can be examined in a simple manner [17]. The test consisted of repeated two 20-m runs at a progressively increased speed controlled by audio bleeps. When the player failed to reach the finishing line in time twice, the distance covered was recorded and represented the test result. 

### 2.4. Match Loads Monitoring

Internal and external responses encountered by players during the match were assessed through 20 Hz portable heart rate (HR) monitors and GPS-units with a frequency of 10 Hz (Polar Team Pro, Polar Electro^®^, Kempele, Finland). After the match, data were downloaded to a computer and analyzed offline using a customized software package. Players’ internal load was recorded during the match using the following metrics: average heart rate (HR) calculated as a percentage of the maximal HR (%HRavg), maximal HR calculated as a percentage of the maximal HR (%HRmax) and the total time (min) spent in five intensity zones (expressed as a percentage of HRmax): 50–59%, 60–69%, 70–79%, 80–89% and >90% of HRmax. Players’ external load was recorded using the following metrics: TD, distances covered at different speed thresholds (3.00–6.99 km/h, 7.00–10.99 km/h, 11.00–14.99 km/h, 15.00–18.99 km/h and >19.00 km/h), average running velocity (Vavg), maximal running velocity (Vmax), number of sprints (>23 km/h), number of total accelerations in different intensity categories (low-intensity: <1.0 m/s^2^; low- to moderate-intensity: 1.0 to 1.9 m/s^2^; moderate- to high-intensity: 2.0 to 2.9 m/s^2^; high-intensity: >3.0 m/s^2^) and number of total decelerations in different intensity categories (low-intensity: <−1.0 m/s^2^; low- to moderate-intensity: −1.0 to −1.9 m/s^2^; moderate- to high-intensity: −2.0 to −2.9 m/s^2^; and high-intensity: >−3.0 m/s^2^).

### 2.5. Statistical Analyses

Normal distribution and homogeneity of variances were tested using the Shapiro–Wilk test and the Levene test, respectively. Differences in internal and external loads between two halves and between pre- and post-match measurements (CMJ, sprint, T-test and YO-YO IR2) were calculated using the paired samples *t*-test. Data are presented as mean (M) ± standard deviation (SD), and differences are also expressed as the percentage change (delta; Δ). Standardized differences based on Hedges’ g effect size (ES) were calculated to analyze the practical significance of match-induced fatigue. ES < 0.2, 0.2–0.5, 0.5–0.8 and >0.8 were considered as trivial, small, moderate and large, respectively [18]. Moreover, a qualitative probabilistic mechanistic inference (90% confidence intervals) was applied to evaluate match-induced fatigue, with inferences based on standardized thresholds for the smallest worthwhile change (SWC), set as 0.2 of the baseline SD [19]. The qualitative probabilistic terms were assigned according to the following thresholds [20]: <0.5%, most unlikely or almost certainly not; 0.5–5%, very unlikely; 5–25%, unlikely or probably not; 25–75%, possibly; 75–95%, likely or probably; 95–99.5%, very likely; and >99.5%, most likely or almost certainly. After that, Pearson’s correlation was used to measure the degree of association of match loads and physical performance measurements. The magnitude of the correlation coefficient was interpreted as follows: ≤0.10, trivial; 0.11–0.30, small; 0.31–0.50, moderate; 0.51–0.70, large; 0.71–0.90, very large; and >0.90, almost perfect. Finally, a backward stepwise regression was applied to find the match loads that best explain the fatigue occurrence after a soccer match. Interpretation of the strength of the relationship based on the R-squared coefficient was carried out using the following scale [21]: <0.3, very weak effect; 0.3–0.5, weak or low effect; 0.5–0.7, moderate effect; and >0.7, strong effect. Correlation and regression analysis were performed using the percentage change (delta; Δ) of both match loads and physical performance measurements. The statistical significance was established at *p* < 0.05. Statistical analyses were performed using the JASP 0.16.3.0 software (University of Amsterdam, Amsterdam, The Netherlands).

## 3. Results

Table 1 present the match-related internal and external loads. A substantial decrease in the time at 90–100% HRmax was only observed in the 2nd half compared with the 1st half. Δ of internal load variables range from 0.09 ± 3.14 (%HRmax) to 76.68 ± 64.24 (time at 50–59% HRmax). Δ of external loads range from −18.33 ± 3.44 (number of sprints) to 7.56 ± 28.3 (TD at 15.00–18.99 km/h).

Neuromuscular performance decrements and intermittent-running endurance capacity impairment immediately post-match are reported in Table 2. The match induced a probable decrease in CMJ height (Δ −9.21 ± 3.12, ES −0.59 [−1.49, 0.30], moderate), whereas decreases were almost certainly observed at post-match both in T-test performance (Δ 7.55 ± 2.93, ES 2.26 [1.14, 3.38], large) and in YO-YO IR2 distance covered (Δ −41.26 ± 9.59, ES −2.43 [−3.58, −1.27], large). No significant differences between pre- and post-match were found in 10-m sprint performance (Δ 3.32 ± 4.85, ES 0.60 [−0.27, 1.52], moderate), but a very likely impairment in 20-m sprint performance was induced by the match loads (Δ 4.73 ± 5.15, ES 0.92 [0.00, 1.84], large). 

The relationships reported in Table 3 demonstrate that some match-loads in particular thresholds most importantly correlate with Δ of CMJ height and 10-m sprint. On one hand, Δ of %HRmax and time at 90–100% HRmax were largely correlated with Δ of 10-m sprint (*r* = 0.636, *p* = 0.048; *r* = 0.660, *p* = 0.038, respectively). On the other hand, Δ of number of high-intensity decelerations and accelerations was largely correlated with Δ of CMJ height (*r* = 0.689, *p* = 0.027; *r* = −0.645, *p* = 0.044, respectively).

The internal and external load metrics that may allow for predicting the match-related fatigue are depicted in Table 4. All internal and external load variables showed a non-significant effect in relation to Δ of CMJ height. Δ of time at 90–100% HRmax (R^2^ = 0.435, weak effect) and different ranges of accelerations and decelerations (R^2^ = 0.888, strong effect) were the main predictors of Δ of 10-m sprint. However, the percentage change of the 20-m sprint may be predicted only from different ranges of accelerations and decelerations (R^2^ = 0.981, strong effect). The T-test performance decrement may be predicted from TD covered at different intensities (R^2^ = 0.813, strong effect), Vavg and Vmean (R^2^ = 0.650, moderate effect) and different ranges of accelerations and deceleration (R^2^ = 0.993, strong effect). Finally, internal load metrics may predict YO-YO IR2 impairment (R^2^ = 0.999, strong effect), but external load variables were not significantly related to the YO-YO IR2 impairment. Internal or external load variables not shown in Table 4 reported non-significant values, thus may not be used for predicting changes in neuromuscular performance decrements and intermittent-running endurance capacity impairment immediately post-match.

## 4. Discussion

The aim of the present study was to determine the associations between soccer match-related internal and external loads, neuromuscular performance decrease and intermittent-running endurance capacity decrement immediately post-match. The main findings indicate that the quantification of Δ of match external-load metrics, particularly accelerations and decelerations, provide a useful non-invasive predictor of subsequent NMF status in soccer players immediately post-match. However, only internal load metrics present practical applications for predicting intermittent-running endurance capacity impairment.

Although muscle fatigue in soccer is considered a multifactorial process with contribution from different fatiguing mechanisms (metabolic, mental, morphological, biochemical, etc.), muscle glycogen depletion has been highlighted as a key factor associated with fatigue late in a game, as well as in determining recovery after a game or an intense training session [2]. Low glycogen in individual muscle fibers and subcellular compartments in the muscle cell is likely to negatively affect several essential steps in the excitation–contraction coupling such as action potential propagation, muscle excitability, calcium handling and cross-bridge cycling through reductions in muscle ATP, which are suggested sites of muscle function impairment inducing muscle fatigue [2]. Consequently, NMF results in decreased force-production capacity concurrent with impairments in the muscle-stretch shortening cycle (SSC) [22].

Measurements of neuromuscular capacity involving SSC, such as a CMJ, have been previously reported to be able to detect fatigue status in soccer players following match play [1]. The scientific literature investigating the effects of a soccer match on CMJ performance showed that performance decreases immediately post-match compared to pre-match (Δ: −5.5%; range: −11.9%, 2.9%; ES: −0.5) [1], which are of a similar magnitude to the current results. However, the CMJ height relationship to match loads has showed confounding results. On one hand, a dose–response relationship was found between CMJ height and low, medium, and high PlayerLoad at 0.5-h post-match [23]. On the other hand, different studies did not find correlations between CMJ height at 0.5-h post-match and external load measurements [3,24]. The current results indicate that Δ CMJ height decrement could be largely linked to Δ of high-intensity decelerations and accelerations, which target the same muscles that are active in CMJ. Differences between the previous results and the current ones may be explained by the outcome metric. Utilizing an outcome metric such as jump height alone may mask the effects of fatigue. Some athletes may alter their jump mechanics when fatigued in order to help maintain jump height [6]. Recent recommendations have suggested that the ratio of flight time to contraction time (FT:CT) may be a more sensitive measure [23], but equipment availability did not allow it to be measured. Notwithstanding, as with previous results [11], we did not find any internal or external load metric that causes NMF to be able to predict CMJ height decrement. Thus, a change in match load could cause either trivial change, or substantial increase or decrease in the jump height. 

Fatigue also involves changes in maximal force, maximal shortening velocity and the curvature of the force–velocity relationship with different underlying mechanisms [25]. In soccer, match-related fatigue is determined by a combination of central and peripheral factors immediately after the match, but central fatigue seems to be the main cause of the decline in sprinting ability [26]. Concretely, peak speed impairment of soccer players during maximal exercise such as sprinting may be explained by a reduction in voluntary activation [26]. Thus, it might be presumed that the significant 20-m sprint impairment observed in the current study may derive from central factors. This reduction in performance is slightly larger than previous findings (Δ: 2.5%; range: 1.4%, 3.5%; ES: 0.8) [1]. However, this 20-m sprint performance impairment is not accompanied by the 10-m sprint time decrease between the baseline and post-match, which agree with previous findings [3]. In contrast to CMJ, how the match-derived load variables relate to post-match sprint performance is scarcely researched. The current results indicate that Δ of 10-m sprint time is largely correlated with some internal loads, in opposition to previous results, which reported no correlations between any of the match-running variables and pre-post changes in measures of the 10- and 20-m sprint [3]. However, the 10- and 20-m sprint performance decrement can be predicted quite accurately using the Δ of accelerations and decelerations in particular thresholds. Intense accelerations and decelerations are particularly related to NMF [27]. Accelerations have a higher metabolic cost compared to decelerations and a greater neural activation of working muscles compared to constant-speed running [28,29,30]. Decelerations have a higher mechanical load [31] that can inflict greater damage on soft-tissue structures especially if high-force impacts cannot be attenuated efficiently [32]. Consequently, these actions may contribute to induced muscle damage, reduced neural drive and mechanical fatigue with potential detrimental effects on performance outcomes [33,34,35,36]. Indeed, the current results lead to a better understanding of the mechanisms underlying why soccer players are fatigued and give insight into NMF which affects sprinting performance.

The high-intensity accelerations and decelerations, commonly sequenced with COD, are crucial for physical performance in soccer. However, the very complex nature of COD performance also relies on straight sprinting speed, reactive strength, technique to change direction, balance, leg muscle properties and anthropometric factors [37,38]. Similarly, the current results show that TD covered at different intensities, Vavg and Vmean and, mainly, accelerations and decelerations are the best predictors of COD performance decrement. Match-induced fatigue results in a moderate decline in COD ability at post-match (Δ: 2.1%; range: 0.2%, 4.2%; ES: 0.7) [1], which is lower than the current findings. This difference in performance decrement may be related to the different COD tests compared, which may have different COD angles. One reason for altered COD performance is the impaired SSC. For example, biomechanical alterations in COD performance following fatigue among soccer players indicate that fatigued athletes may achieve less knee flexion and greater hip flexion, which decrease the ability to utilize SSC [39]. Thus, it is not surprising that Δ of accelerations and decelerations between halves may predict fatigue occurrence accurately during a COD test, because the acceleration–deceleration dynamics associated with COD require high levels of mechanical (e.g., eccentric contractions) load [29]. The influence of high-intensity decelerations during match play is of particular note as high eccentric braking actions seem to account for the highest magnitude of mechanical load per meter than any other match play locomotor activity [31]. Notwithstanding, the current findings indicate that sprint speed decrement during match play is also an important predictor of the impairment of the player’s ability to change direction. This result is in agreement with previous studies. Moderate correlations have been observed between COD performance and linear sprint times [37], which is able to explain 58% of the variance in a COD test [40]. 

The current results confirm that match-related fatigue also induces an intermittent-running endurance capacity impairment. This was corroborated using the YO-YO IR2 test, a simple method for examining an athlete’s ability to perform intense intermittent exercise with a high aerobic energy production and a significant contribution of the anaerobic energy system [8]. Considering the muscle glycogen utilization reported during the YO-YO IR2 test [17], and given the determinants’ YO-YO test performances [8,41], it may be inferred that these decreases result from the abovementioned match-induced mechanical (e.g., force production) and metabolic (e.g., glycogen stores) impairments. Interestingly, all internal load metrics were significant predictors of the YO-YO IR2 test impairment, whereas external load variables failed to know the probability of YO-YO IR2 test impairment. This may be supported by preceding findings which indicate that YO-YO IR2 performance correlates to VO2max (*r* = 0.56) but not to 30- and 50-m sprint performance [17].

The sample size and the level of participants are some of the limitations of the study. Moreover, velocity and distance metrics are frequently reported measures of external load, yet they may underestimate the load in sports such as soccer where frequent changes of velocity and direction occur [42]. Actions such as kicking, jumping and heading are also powerful movements that are repeatedly performed by players during competitive soccer, and may contribute to non-metabolic fatigue of the muscular and physiological system, yet they are rarely considered as contributors to post-match fatigue [43]. Unfortunately, they were unable to be measured. Additionally, the rating of perceived exertion (RPE) is one of the most common means of assessing internal load, but it was not measured. Its monitoring may also provide information regarding the player’s physiological stress during matches. Finally, we did not use individualized load thresholds, which can more efficiently reflect the acute post-match fatigue. Thus, future studies should include different level and gender players, larger samples and a broad range of match-related metrics and fatigue measurements to confirm the current findings. Caution is necessary, however, when making inferences from data derived in studies investigating populations of differing playing standards. The difference in activity patterns between official matches and friendly matches in male soccer players should also be considered when making comparisons [44].

## 5. Conclusions

The relationships between match activity variables and fatigue status show that the quantification of Δ of match external-load metrics, particularly accelerations and decelerations, provide a useful non-invasive predictor of subsequent NMF status in soccer players immediately post-match. However, only internal load metrics present practical applications for predicting intermittent-running endurance capacity impairment.

Based on an applied perspective, multiple factors need to be considered when assessing fatigue status given the lack of evidence related to the use of single measures of fatigue status [45]. In addition, most previous studies have analyzed the associations between match loads and fatigue markers using absolute values, but it seems more appropriate to compare players to themselves, because an estimation of how the players themselves evolve would provide a simpler and more valid approach to determine the reference ranges to be used to guide interpretations. Therefore, variability between halves may represent a valuable alternative to facilitate the analysis of match-related fatigue both for research and applied purposes.

## Figures and Tables

**Table 1:** Match-related internal and external loads.

	1st Half (M + SD)	2nd Half (M + SD)	Δ (% Change)
**Internal loads**			
%HRavg	78.5 ± 6.7	75.1 ± 7.22	−4.15 ± 7.2
%HRmax	94.1 ± 5.2	94.2 ± 6.27	0.09 ± 3.14
Time (min) at 50–59% HRmax	1.46 ± 2.58	2.57 ± 3.03	76.68 ± 64.24
Time (min) at 60–69% HRmax	6.85 ± 4.39	8.21 ± 6.69	17.5 ± 80.56
Time (min) at 70–79% HRmax	13.56 ± 5.64	14.33 ± 3.82	13.91 ± 24.81
Time (min) at 80–89% HRmax	16.75 ± 5.8	15.66 ± 7.23	−2.22 ± 40.2
Time (min) at 90–100% HRmax	6.28 ± 8.22	4.24 ± 6.15 *	−32.68 ± 64.8
**External loads**			
TD (m)	5074.26 ± 462.27	4816.29 ± 484.68	−4.47 ± 11.74
TD (m) at 3.00–6.99 km/h	2072.09 ± 296.53	1966.03 ± 129.12	−3.62 ± 12.57
TD (m) at 7.00–10.99 km/h	1524 ± 221.87	1440.03 ± 213.22	−4.38 ± 14.47
TD (m) at 11.00–14.99 km/h	935.08 ± 209.51	852.05 ± 271.28	−9.09 ± 19.8
TD (m) at 15.00–18.99 km/h	369 ± 98.71	389.08 ± 119.30	7.56 ± 28.3
TD (m) at >19.00 km/h	174.09 ± 84.77	169.1 ± 115.24	−1.2 ± 65.46
Vavg	6.68 ± 0.43	6.36 ± 0.46	−4.54 ± 7.82
Vmax	25.44 ± 1.14	24.88 ± 1.24	−2.17 ± 3.4
No. sprints (>23 km/h)	5.4 ± 2.59	4.4 ± 2.46	−18.33 ± 3.44
No. high-intensity DEC (>−3.0 m/s^2^)	32.9 ± 5.86	32.8 ± 4.69	1.43 ± 18.29
No. moderate to high-intensity DEC (−2.9 to −2.0 m/s^2^)	65.7 ± 12.36	65.5 ± 12.15	−0.08 ± 6.39
No. low to moderate DEC (−1.9 to −1.0 m/s^2^)	83.2 ± 16.47	76.6 ± 9.59	−6.35 ± 11.12
No. low DEC (−0.9 to −0.5 m/s^2^)	210 ± 13.82	205.3 ± 12.64	−2.13 ± 4.38
No. low ACC (0.5 to 0.9 m/s^2^)	199.2 ± 19.76	195.6 ± 14.70	−1.53 ± 4.93
No. low to moderate ACC (1.0 to 1.9 m/s^2^)	121.3 ± 26.24	114.9 ± 18.98	−3.9 ± 10.55
No. moderate to high-intensity ACC (2.0 to 2.9 m/s^2^)	66.3 ± 8.42	64.1 ± 7.09	−2.83 ± 8.09
No. high-intensity ACC (>3.0 m/s^2^)	31.5 ± 5.93	30.5 ± 5.02	−2.64 ± 5.64

ACC: accelerations; DEC: decelerations; HRmax: maximum heart rate; HRavg: average heart rate; TD: total distance covered; Vmax: maximal velocity; Vavg: average velocity. * Statistically significant difference (*p* < 0.05).

**Table 2:** Mean differences, standardized differences and qualitative probabilistic mechanistic inference of neuromuscular performance and intermittent-running endurance capacity.

	Pre-Match	Post-Match	Δ (% Change)	*p*	ES (95% CI)	ES Magnitude	Probabilistic Inference
**CMJ height (cm)**	36.71 ± 5.18	33.4 ± 5.42 *	−9.21 ± 3.12	0.000	−0.59 (−1.49, 0.30)	Moderate	Probably
**10-m sprint (s)**	1.8 ± 0.07	1.86 ± 0.11	3.32 ± 4.85	0.058	0.62 (−0.27, 1.52)	Moderate	Probably
**20-m sprint (s)**	3.12 ± 0.08	3.26 ± 0.19 *	4.73 ± 5.15	0.018	0.92 (0.00, 1.84)	Large	Very likely
**T-test (s)**	9.33 ± 0.21	10.04 ± 0.37 *	7.55 ± 2.93	0.000	2.26 (1.14, 3.38)	Large	Almost certainly
**YO-YO IR2 (m)**	648 ± 102.93	384 ± 105.32 *	−41.26 ± 9.59	0.000	−2.43 (−3.58, −1.27)	Large	Almost certainly

CI: confidence interval; CMJ: countermovement jump; ES: effect size; YO-YO IR2: Yo-Yo intermittent recovery test level 2. * Statistically significant difference (*p* < 0.05).

**Table 3:** Correlations between percentage change (Δ) of match-related loads and percentage change (Δ) of neuromuscular performance and intermittent-running endurance capacity.

	*r*	*r* Magnitude	*p*
Δ 10-m sprint and Δ %HRmax	0.636	Moderate	0.048
Δ 10-m sprint and Δ time (min) at 90–100% HRmax	0.660	Moderate	0.038
Δ CMJ height (cm) and Δ No. high-intensity DEC (>−3.0 m/s^2^)	0.689	Moderate	0.027
Δ CMJ height (cm) and Δ No. high-intensity ACC (>3.0 m/s^2^)	−0.645	Moderate	0.044

ACC: accelerations; CMJ: countermovement jump; DEC: decelerations; HRmax: maximum heart rate; TD: total distance covered; Δ: percentage change.

**Table 4:** Values of multiple linear regression with backward stepwise elimination explaining the contribution of internal and external loads to the percentage change (Δ) of fatigue measurements. Only statistically significant models are reported.

Dependent Variable	Model				Independent Variable	B	SE	β	*p*	VIF
	R	R^2^	Adjusted R^2^	F						
**Δ 10-m sprint** **(s)**	0.660	0.435	0.365	6.165	**Internal load:**					
					Δ Time (min) at 90–100% HRmax	0.049	0.020	0.660	0.038	1.000
	0.943	0.888	0.749	6.368	**External load (ACC and DEC):**					
					Δ No. moderate to high-intensity DEC (−2.9 to −2.0 m/s^2^)	0.510	0.158	0.673	0.032	1.563
					Δ No. low to moderate DEC (−1.9 to −1.0 m/s^2^)	−0.450	0.105	−1.031	0.013	2.067
					Δ No. high-intensity ACC (>3.0 m/s^2^)	−1.104	0.258	−1.285	0.013	3.230
**Δ 20-m sprint** **(s)**	0.990	0.981	0.943	25.675	**External load (ACC and DEC):**					
					Δ No. moderate to high-intensity DEC (−2.9 to −2.0 m/s^2^)	1.078	0.142	1.337	0.005	4.860
					Δ No. low to moderate DEC (−1.9 to −1.0 m/s2)	−0.614	0.084	−1.324	0.005	5.103
					Δ No. low DEC (−0.9 to −0.5 m/s^2^)	0.513	0.140	0.437	0.035	2.220
					Δ No. low ACC (0.5 to 0.9 m/s^2^)	0.868	0.171	0.829	0.15	4.198
					Δ No. high-intensity ACC (>3.0 m/s^2^)	−1.687	0.265	−1.846	0.008	13.190
**Δ T-test** **(s)**	0.902	0.813	0.719	8.692	**External load (distances):**					
					Δ TD (m) at 3.00–6.99 km/h	−0.222	0.052	−0.955	0.005	1.619
					Δ TD (m) at 15.00–18.99 km/h	0.147	0.035	1.424	0.006	3.698
					Δ TD (m) at >19.00 km/h	−0.035	0.014	−0.789	0.048	3.269
	0.806	0.650	0.550	6.510	**External load (velocity):**					
					Δ Vavg	0.428	0.122	1.144	0.010	2.127
					Δ Vmax	−0.877	0.280	−1.019	0.017	2.127
	0.996	0.993	0.978	67.532	**External load (ACC and DEC):**					
					Δ No. moderate to high-intensity DEC (−2.9 to −2.0 m/s^2^)	0.583	0.050	1.274	0.001	4.860
					Δ No. low to moderate DEC (−1.9 to −1.0 m/s^2^)	−0.410	0.029	−1.558	<0.001	5.103
					Δ No. low DEC (−0.9 to −0.5 m/s^2^)	−0.237	0.049	−0.355	0.017	2.220
					Δ No. low ACC (0.5 to 0.9 m/s^2^)	0.976	0.060	1.643	<0.001	4.198
					Δ No. moderate to high-intensity ACC (2.0 to 2.9 m/s^2^)	0.625	0.046	1.727	<0.001	6.486
					Δ No. high-intensity ACC (>3.0 m/s^2^)	−1.531	0.093	−2.951	<0.001	13.190
**Δ YO-YO IR2** **(m)**	0.999	0.999	0.995	239.936	**Internal loads**					
					Δ %HRavg	0.722	0.069	0.579	0.008	4.497
					Δ %HRmax	−2.900	0.173	−0.948	0.004	5.396
					Δ Time (min) at 50–59% HRmax	0.027	0.001	1.706	0.002	7.613
					Δ Time (min) at 60–69% HRmax	−0.300	0.008	−2.517	<0.001	8.025
					Δ Time (min) at 70–79% HRmax	−0.155	0.012	−0.402	0.006	1.544
					Δ Time (min) at 80–89% HRmax	−0.075	0.013	−0.315	0.030	5.272
					Δ Time (min) at 90–100% HRmax	−0.209	0.006	−1.412	<0.001	3.098

ACC: accelerations; B: unstandardized regression coefficients; β: standardized regression coefficients; DEC: decelerations; HRavg: average heart rate; HRmax: maximum heart rate; SE: standard error of the regression coefficients; TD: total distance covered; Vavg: average velocity; VIF: variance inflation factor; Vmax: maximal velocity; YO-YO IR2: Yo-Yo intermittent recovery test level 2; Δ: percentage change.

## Data Availability

Not applicable.
